# Divergence of protein-coding capacity and regulation in the *Bacillus cereus sensu lato *group

**DOI:** 10.1186/1471-2105-15-S11-S8

**Published:** 2014-10-21

**Authors:** Inimary T Toby, Jonah Widmer, David W Dyer

**Affiliations:** 1 University of Oklahoma Health Sciences Center, 975 NE 10th Street, BRC-1106, Oklahoma City, OK 73104, USA

**Keywords:** Prokaryotic Evolution, comparative, divergence, sigB, B. cereus sensu lato

## Abstract

**Background:**

The *Bacillus cereus sensu lato *group contains ubiquitous facultative anaerobic soil-borne Gram-positive spore-forming bacilli. Molecular phylogeny and comparative genome sequencing have suggested that these organisms should be classified as a single species. While clonal in nature, there do not appear to be species-specific clonal lineages, excepting *B. anthracis*, in spite of the wide array of phenotypes displayed by these organisms.

**Results:**

We compared the protein-coding content of 201 *B. cereus sensu lato *genomes to characterize differences and understand the consequences of these differences on biological function. From this larger group we selected a subset consisting of 25 whole genomes for deeper analysis. Cluster analysis of orthologous proteins grouped these genomes into five distinct clades. Each clade could be characterized by unique genes shared among the group, with consequences for the phenotype of each clade. Surprisingly, this population structure recapitulates our recent observations on the divergence of the generalized stress response (SigB) regulons in these organisms. Divergence of the SigB regulon among these organisms is primarily due to the placement of SigB-dependent promoters that bring genes from a common gene pool into/out of the SigB regulon.

**Conclusions:**

Collectively, our observations suggest the hypothesis that the evolution of these closely related bacteria is a consequence of two distinct processes. Horizontal gene transfer, gene duplication/divergence and deletion dictate the underlying coding capacity in these genomes. Regulatory divergence overlays this protein coding reservoir and shapes the expression of both the unique and shared coding capacity of these organisms, resulting in phenotypic divergence. Data from other organisms suggests that this is likely a common pattern in prokaryotic evolution.

## Backgound

The *Bacillus cereus sensu lato *group contains a variety of facultative anaerobic soil-borne Gram-positive spore-forming bacilli that are ubiquitous in nature. This group consists of at least seven species (*B. cereus, B. anthracis, B. mycoides, B. pseudomycoides, B. thuringiensis, B. weihenstephanensis *and *B. cytotoxicus*) that new molecular phylogenies and comparative genome sequencing have suggested should be classified as a single species [1]. These various species are phylogenetically interspersed among one another in a variety of phylogenies. While clonal in nature, there do not appear to be species-specific clonal lineages in this assemblage, with the exception of the *B. anthracis *lineage [2]. Paradoxically, these organisms display a wide array of biological behaviors, despite their close taxonomic and phylogenetic relationship. *B. anthracis *is the cause of the acute and often lethal disease anthrax that can infect a wide variety of mammalian hosts, with differing virulence characteristics [3]. *B. thuringiensis *is a useful source of insecticidal toxins, often in the form of spore-containing preparations of crystal protein toxins (*cry *toxins); however, some *B. thuringiensis *strains have been isolated from severe human infections [4-6]. *B. cereus *is often isolated as an opportunistic pathogen, and causes contamination problems in the dairy industry and paper mills [7]. Food poisoning isolates rarely are associated with invasive disease, and appear predominantly to cause enterointoxication without overt colonization. Other *B. cereus *strains are part of the normal gut microflora [4]. *B. weihenstephanensis *is a psychrophile, as are several *B. cereus *strains suggested to actually be *B. weihenstephanensis *[8]. Recent observations have suggested the addition of two additional species to the *B. cereus sensu lato *group, tentatively designated as *B. gaemokensis *and *B. manlipoensis *[9]. However, little is currently known about the genomic basis for the taxonomic affiliation of these newly described organisms.

Studies seeking to understand the underlying basis of these varied phenotypes have often focused on the complement of extrachromosomal elements harbored by these organisms, with good reason [10]. The capacity to cause invasive toxigenic disease by *B. anthracis *is intimately rooted in the presence of the anthracis virulence plasmids pXO1 and pXO2 [11]. Similarly, the only clearly established differences between *B. cereus *and *B. thuringiensis *strains is the presence of genes coding for the *cry *toxins, typically plasmid-borne, although sometimes chromosomally encoded [6]. If the *cry *plasmids are lost, *B. thuringiensis *can no longer be distinguished from *B. cereus *[4]. The origins of virulent anthrax-like strains from multiple nonpathogenic ancestors offer an opportunity to better understand the origins of pathogenicity in this *Bacillus *group. It has been proposed that pathogens in this group have high virulence potential, but that their origin is limited solely by the chance horizontal gene transfer (HGT) of toxin-expressing plasmids [11]. Alternatively, it has also been proposed that multiple historical origins of pathogenic strains have occurred because of existing pre-adaptations or newly arising adaptive changes in the genomes of nonpathogenic ancestors [12]. The reconciliation of contradictory relationships exhibited by members of this phylogenetic group of species is still ongoing [10]. Some studies have clearly demonstrated that chromosomal-encoded determinants, such as *plcR *and enterotoxins play an important role in biological behavior [13]. Zwick *et al*. examined pathogenic and non-pathogenic *B. cereus sensu lato *draft and whole genome sequences to test whether *B. anthracis *biovar *anthracis *CI, or other similar strains were unique in terms of the gain or loss of specific genes or whether they had DNA signatures suggestive of a newly emerged pathogen. They found that little evidence for adaptive changes in the *B. anthracis *genome that uniquely predispose it for a virulent lifestyle [12]. In a related study, Papazisi *et al*. utilized comparative genome hybridization using DNA microarrays to gain insights into the unique genomic features encoded in the *B. anthracis *genome in comparison to other *B. cereus *group members. They identified genomic events associated with the emergence of *B. anthracis *as a distinct lineage within the *B. cereus *group [10]. However, despite several studies [14,15], the mechanisms leading to the evolution and emergence of pathogenesis in the *B. cereus sensu lato *group remain unclear.

Our intent is to begin to deduce the phenomic differences between these organisms, beginning with genome sequence data. Although 'phenomics' would at first appear to be yet another of the '-omics' terms that has been coined in the last 10-15 years, the term 'phenome' was originally coined in 1949 [16], long before the current '-omics' revolution began. The phenome is essentially information that describes the total phenotypic potential of the organism, and is dependent on all other components of the organism, including the genome sequence, the regulatory relationships encoded in the genome, etc. For example, discussions led by A.Varki among those who had used the term up to 2003 suggested the following definition: "The body of information describing an organism's phenotypes, under the influences of genetic and environmental factors" [17]. Thus, our initial goal in this study was to begin to define the collective contribution of the chromosomally-encoded protein set of a given genome to the phenotypic variation in these organisms.

## Materials and methods

### Genomes and Annotations used in this study

201 draft and whole genome sequences of the *Bacillus cereus sensu lato *group were collected (Additional file 1). From this list, we parsed out 25 whole genome sequences and submitted them as a fasta file to the University of Maryland Institute for Genome Sciences (UMd) for re-annotation using their IGS analysis engine [18]; subsequently, these will be referred to as the UMd annotations. These re-annotations were received on January 20 and October 8, 2012 respectively (Table 1). Our database was frozen after October 8 so that this resource would remain stable for this analysis; whole genomes that appeared after this point were excluded for this practical reason.

**Table 1:** Whole genomes used in this study

Genome	Locus Tag	NCBI Accession #	UMd identifier	Annotation Date	Sequencing group
**B. anthracis A0248**	BAA	NC_012659.1	bac1	04/2005	LANL
**B. cereus biovar anthracis CI**	BACI	NC_014335.1	bac2	06/2006	GGL
**B. anthracis Ames**	BA	NC_003997.3	bac3	02/1999	TIGR
**B. thuringiensis Al. Hakam**	BALH	NC_008600.1	bac4	10/2002	JGI
**B. anthracis CDC 684**	BAMEG	NC_012581.1	bac5	03/2005	LANL
**B. anthracis Sterne**	BAS	NC_005945.1	bac6	12/1999	JGI
**B. cereus AH187**	BCAH187	NC_011658.1	bac7	11/2004	JCVI
**B. cereus AH820**	BCAH820	NC_011773.1	bac8	09/2004	JCVI
**B. cereus B4264**	BCB4264	NC_011725.1	bac9	11/2004	JCVI
**B. cereus ATCC 10987**	BCE	NC_003909.8	bac10	08/2000	TIGR
**B. cereus ATCC 14579**	BC	NC_004722.1	bac11	02/1999	INRAGM
**B. cereus G9842**	BCG9842	NC_011772.1	bac12	09/2004	JCVI
**B. cereus Q1**	BCQ	NC_011969.1	bac13	12/2004	MGCC
**B. thuringiensis BMB171**	BMB171	NC_014171.1	bac14	04/2006	HAU
**B. weihenstephanensis- KBAB4**	BcerKBAB4	NC_010184.1	bac15	04/2006	HAU
**B. cereus 03BB102**	BCA	NC_012472.1	bac16	02/2005	LANL
**B. cereus E33L**	BCZK	NC_006274.1	bac17	10/2000	JGI
**B. thuringiensis serovar- konkukian str 97-27**	BT9727	NC_005957.1	bac18	11/2000	JGI
**B. anthracis Ames Ancestor**	GBAA	NC_007530.2	bac19	02/1999	TIGR
**B. cytotoxicus NVH391-98**	Bcer98	NC_009674.1	bac 20	06/2003	JGI
**B. anthracis H9401**	BAH9401	NC_017729.1	bac 21	06/2012	KCDC
**B. cereus F837/76**	BF83776	NC_016779.1	bac 22	06/2012	INRAGM
**B. cereus NC7401**	BNC7401	NC_016771.1	bac 23	09/2012	KILS
**B. thuringiensis serovar- chinensis CT43**	BCT43	NC_017208.1	bac 24	09/2012	HAU
**B. thuringiensis serovar-finitimus YBT-020**	BYBT020	NC_017200.1	bac 25	09/2012	HAU

Dates/location of original annotations. Full name for sequencing group are as follows: LANL: Los Alamos National Labs, JCVI: J. Craig Venter Institute, INRAGM: INRA Genetique Microbienne, MGCC: Microbial Genome Center of Chinese Ministry of Public Health, HAU: Huazhong Agricultural University, GGL: Goettingen Genomics Laboratory, UMd: University of Maryland Institute for Genome Sciences, KCDC: Korea Center for Disease Control, KILS: Kitasato Institute for Life Sciences

### Orthologous protein clustering and hierarchical genome clustering

Clusters of putative orthologous proteins were generated for the 201 draft and whole genomes from NCBI. From this group of genomes, we selected a subset consisting of 25 whole genomes and generated clusters of orthologous proteins from the group of 25 original annotations available at NCBI and for the 25 UMd annotations, using CD-hit [19] with a cutoff of 85/85. In our experience, CD-hit offered comparable results to other orthology search programs such as OrthoMCL [20] and was computationally much faster. Similar to previously described methods performed by our group [21], this cutoff required an 85% sequence identity across 85% sequence length for CD-hit while all other parameters remained at the default values. Manually inspecting this output, we confirmed that a representative protein from each organism was grouped with other similar proteins to form the orthologous clusters. From the CD-hit output, two separate datasets were generated. The first one had a total of 92,806 clusters with sizes ranges from 1-201 genomes; the second dataset had a total of 21,288 clusters with cluster sizes ranging from 1-50 genes. For the 21,288 clusters, the total number of genes comprising the core genomes was calculated using all clusters ranging from 25-50 genes in size. Files were extracted and stored in a MySQL database. Next, for both sets of cd-hit outputs, orthologous clusters were parsed out for each organism and separate tables were created using Perl scripts. These tables were then imported into an excel spreadsheet and calculations were done for the presence (assigned a 1) or absence (assigned a 0) of genomes within a specific cluster using Visual Basic/macros coding. The final file was uploaded into Matlab. We then applied the 'Heatmap' and 'clustergram' scripts using Matlab [22] to render a 2-d color image of the data showing the cluster numbers on the y-axis and the organisms on the x-axis. To organize this data and identify potential relationships among the genome-specific orthologous clusters, we utilized a hierarchical clustering with Euclidean distance metric and average linkage to generate the hierarchical tree. This type of clustering enabled us to find the similarity or dissimilarity between every pair of objects in the data set, group the objects into a binary, hierarchical cluster tree, and determine where to differentiate the hierarchical tree into clusters.

### Clusters of Orthologous Genes (COGS) analysis

We extracted all clusters from the whole genomes dataset that contained 24 and 25 members of orthologous proteins with one representative member from each organism as derived from CD-hit analysis. The 24 member clusters allowed us to include clusters with and without *B. cytotoxicus *as it was an outlier from our previous analysis. This yielded 2,674 protein sequences total for COGs analysis. We generated a list of proteins found in each organism per a given clade. Using these individual lists, we then proceeded to extract all the nucleotide sequences from the genes using Perl scripts to create new fasta files. We then imported these fasta sequences from NCBI and performed blastX. The blastX output files were extracted, saved, and uploaded into MEGAN v 5_1_4 [23] for COGs analysis. MEGAN was only able to successfully run ~200 genes at one time so we separated the genes into several lists and performed multiple runs to generate the COGS data. We selected the COGS analysis feature in MEGAN and then selected the file type we were using. The output generated by MEGAN showed a tree with the biological categories and our genes with respective ids located within the branches. These calculations and raw data were then extracted and saved as text files.

### Metabolic pathway analysis

The original (NCBI) and UMd annotations for each of the 25 *B. cereus sensu lato *genomes were uploaded into an in-house Pathway Tools database [24]. Pathway predictions were then made based on these annotations for each genome using SRI's software algorithm. This data was downloaded, and organized into an external database for each organism. KEGG global metabolic maps [25] were constructed using the KEGG identifiers for enzymes located in the KEGG database. The KEGG database enzyme list was downloaded and saved as a csv file. Enzyme lists for each *B. cereus sensu lato *clades were parsed from the database into a csv file. These two lists were then compared using Visual Basic/macros coding. We then uploaded this information into the KEGG MAPPER and utilized the advance pathway mapping utility feature to search and color the individual enzymes. Global comparisons of total protein coding content and metabolic pathway predictions were compared and statistically evaluated using GraphPad Prism v.4.03 [26].

### Analysis of conserved/hypothetical proteins

We examined potential structure features of the conserved hypothetical and hypothetical proteins from our whole genomes dataset. Conserved/hypothetical protein sequences were extracted from a master file using a Perl script. Each ID and sequence were paired in FASTA format and saved to separate files for each of the four clades. The sequences in all four clades were then compared to the Pfam database [27], and analyzed using SignalP [28] and TmHmm [28]. Raw data from the Pfam database was evaluated based on three stringency levels [27]. The most significant hits were those with e-values of <0.001, followed by e-values between 0.1-0.001, and lastly those with e-values higher than 0.1. Pfam also provided assigned family names and clan numbers where available. All fields were kept at default values for the SignalP searches with the exception that the organism field was selected as "gram-positive bacteria". The TmHmm output format chosen was "extensive with graphics" [28]. All data generated from Pfam, SignalP, TmHmm were extracted and saved as text files. All graphical data generated from SignalP and TmHmm were also extracted and saved as individual files. For additional analysis the text files were then converted to excel spreadsheets and Visual Basic scripts were utilized to parse the raw data to create summaries of the findings for each clade.

## Results and discussion

### Whole-genome comparisons of protein-coding content suggest that the B. cereus sensu lato organisms comprise four distinct clades

**Figure 1:**
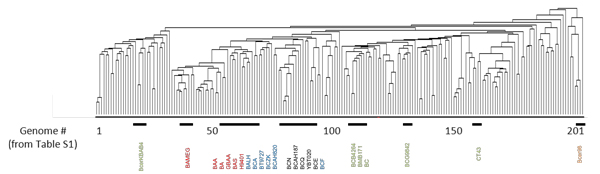
**Orthologous protein cluster comparison of 201 whole and draft B**. Cereus Sensu lato organisms highlights specific clades. Clustergram was derived using hierarchical clustering with Euclidean distance. The full list of genomes and the order for which they appear on the clustergram can be found in Additional file 1. The relative locations and clade designations of the 25 whole genomes from Figure 2 have been labeled on the x-axis of this figure. Genome names colored in red denote Clade C, blue denote Clade D, green denote Clade B, black denote Clade A and orange denote Clade E.

**Figure 2:**
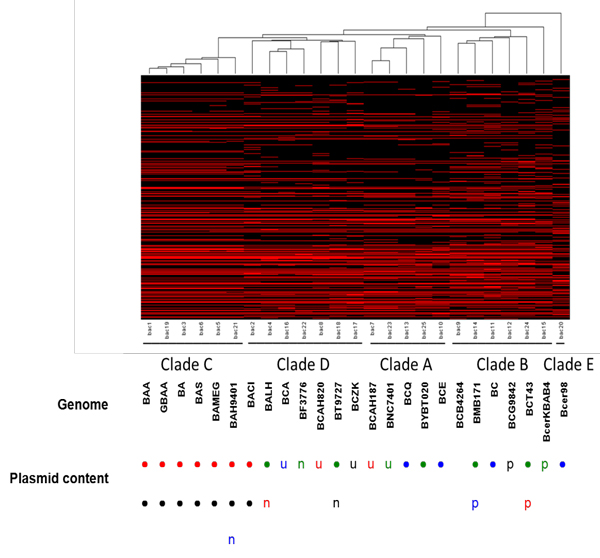
*B. cereus sensu lato *organisms form 5 distinct clades. Clade structure derived using hiearchical clustering with Euclidean distance. 5 clades were derived, Clades A-E. Plasmids found in each organism are denoted by the following: •-pXO1, •-pXO2, •-cry toxin, •-pBC, u-pAH, n-pBT, p-pBMB, p-pCT, n-pF837, n-BAP, u-pNC, u-pE33, u-p03BB102, n-pALH, p-pBWB, p-pG9842.

Our first analysis employed the dataset of all available *B. cereus sensu lato *genomes (draft and whole genomes, Additional file 1). These genomes were analyzed for shared orthologous protein clusters using Cd-hit [18]. As will be discussed below, we parsed the completed genomes for a separate analysis. In each instance, this was followed by performing a Hierarchical Euclidean cluster analysis [22], expressing the results in a simple heatmap and dendrogram (Figure 1). This analysis clustered potential contributors to the phenomotypic differences between these organisms, by virtue of the phenomotypic effectors (proteins) that each genome harbored. Analysis of the dataset, consisting of 201 whole and draft genomes was illustrated by, a dendrogram, a tree-structured graph used in heat maps to visualize the result of the hierarchical clustering calculation (Figure 1, Additional files 2 and 3). Here we show the row dendrogram which illustrates the distance or similarity between rows and which nodes each row belongs to, as a result of clustering [22]. On the x-axis, we show the relative location of 25 whole genomes within this larger group of genomes (Figure 1, Additional file 1). From this analysis, patterns of the relationships between these genomes (see below) and their phenotypic potential began to emerge. However, these patterns were not as clear-cut as could be discerned from the comparisons of the completed genomes alone (see below and Figure 2), likely for several reasons. Since majority (almost 90%) of these genomes were draft sequences, the variable quality of these sequences and their annotation could be complicating factors. For instance, lack of a protein coding sequence in a draft genome annotation could be due to deletion of this coding sequence from the genome, lack of sampling of this region during the sequencing for the draft assembly, low sequence quality that may have excluded certain proteins from a given orthologous cluster, or differences in annotation. The annotations associated with these genomes may be somewhat uneven between sequencing groups, likely due in part to the different annotation methods and sequencing centers from which these data were obtained (e.g., see below for annotation comparisons of the completed genomes). And, for at least 10% of the genomes that we analyzed, the sequencing center provided no information describing the source or origin of these isolates (see Additional file 1). Associating these cryptic-source strains with a specific biological phenomotype was essentially impossible. In the face of these potential and real sources of variation, we selected a subset of these strains (all complete genome sequences) for separate analysis, and for comparison back to the total genome dataset. We also reannotated these whole genome sequences to provide a uniform annotation that eliminated much of the variability that may have affected the cluster analysis shown in Figure 1. The analysis of the whole genomes where we looked at the presence/absence of a particular orthologous cluster was informative, even if that cluster was comprised of proteins annotated as conserved hypothetical or hypothetical proteins (Figure 2, Additional file 4). In this cluster analysis, the heatmap shown is highlighting the presence of a genome within a particular orthologous protein cluster which was assigned a 1 (red in Figure 2), while the absence of a genome within a cluster was assigned a 0 (black in Figure 2). Genomes are represented by the x-axis while the clusters are represented by the y-axis (0-21,288 clusters starting from the bottom to top). Excluding *B. cytotoxicus *(as noted above), we found four distinct clades from this cluster analysis, designated clades A-D (the rationale for these clade designations will be presented below); this data is summarized in Table 2. When we identified the position of these completed genomes in the clustergram shown in Figure 1, these genomes were clustered together similarly in both clustergrams (compare Figure 1 and 2). This suggests that in general, the biological relationships (see next) deduced from the clustergram and heatmap shown in Figure 2 also reflects the larger dataset shown in Figure 1.

**Table 2:** Differences in protein coding capacity mirror the stress response of the organism.

**Genome clusters (from **Figure 2**)**	Organisms	Source or location of isolation	SigB clade (from Reference #19)	SigB regulon constituents
**Clade A**	BCB4264	Bloodstream of pneumonia patient	A	Core SigB regulon

	BMB171	Soil	A	regulatory proteins,

	BC	Dairy product	A	cardiolipin biosynthesis,

	BCG9842	Stool sample from food poisoning outbreak	A	efflux pumps, SOS

	BCT43	insecticide	N/A	functions

	BcerKBAB4	Soil	A	

**Clade B**	BCAH187	Dairy product	B	Core SigB regulon only

	BNC7401	Food poisoning	N/A	

	BCQ	Deep oil reservoir	B	

	BYBT020	insecticide	N/A	

	BCE	Cheese spoilage	B	

**Clade C**	GBAA	Bovine carcass	C	Core SigB regulon

	BA	Bovine carcass	C	regulatory proteins,

	BAA	Human disease	C	S-layer protein,

	BAS	Vaccine strain	C	GalNac biosynthesis,

	BAH9401	Human disease	C	spore germination protein

	BAMEG	CDC isolate	C	

**Clade D**	BACl	Chimpanzee carcass	D	Core SigB regulon

	BALH	Iraq bioweapons facility	D	other additions

	BCA	Human blood isolate	D	similar to SigB Clade C

	BF83776	Human prostate wound isolate	N/A	

	BCAH820	Human periodontitis	D	

	BT9727	Human tissue necrosis	D	

	BCZK	Zebra carcass	D	

Comparison of our clade organisms to previously published clade structure from SigB. N/A denotes genome sequences not available at time of sig B analysis. Previously we have shown that the clades listed correspond to differences in stress responses of sigma factor B across the *B. cereus sensu lato *group of organisms. These differences in stress response also correlate with protein coding capacity of these organisms.

Data from Figure 2 suggests that Clade A appears to be primarily comprised of organisms that were either environmental isolates or associated with food poisoning outbreaks, although in one case, a Clade A organism was isolated from a case of human septicemia. Clade B organisms appear to be derived from either soil or food poisoning episodes, and may represent a group with the least potential to cause serious mammalian disease, In general, Clades A and B appear less capable of initiating invasive infections than organisms found in Clades C and D. This impression that the four clades identified in Figure 2 correlate well with mammalian virulence potential is reinforced by a comparison with the presence of plasmid DNAs harbored in these organisms (lower half of Figure 2). As noted above, with the exception of the pXO1/pXO2 plasmids found in Clade C and the single *B. cereus *biovar *anthracis *Cl strain in Clade D, there is no clear pattern to the relationship between plasmid content and the mammalian virulence potential of the clades shown in Figure 2.

Clade C consisted solely of *B. anthracis *strains. As with many other comparative analyses that have been performed [12], these organisms appear to be comprise a clonal group easily separable from other *B. cereus sensu lato *organisms. While the pXO1 and pXO2 plasmids that encode these virulence factors in *B. anthracis *strains are clearly critical for the ability to cause septicemic disease, the observation that aggregate genomic protein coding content of Clade C organisms clusters separately from other *B. cereus sensu lato *organisms argues that other components of the genome of this organism also participate in the unique biology of the anthrax organism. Similarly, Clade D organisms were typically isolated from episodes of invasive disease. Indeed, one of these, *B. cereus *biovar *anthracis *Cl (isolated from a chimpanzee carcass), was shown to carry the anthrax-associated plasmids pXO1 and pXO2 [11]. This organism was part of a group of closely related isolates that caused deadly anthrax-like infections in primates in the Côte d'Ivoire in 2001-2002 and Cameroon in 2004 [29]. Other clade D organisms include a strain isolated from a case of invasive human disease, (*B. cereus *03BB102) that did not harbor the pXO1/pXO2 virulence plasmids. Clade D also includes a human tissue necrosis isolate (*B. thuringiensis *konkukian 97-17) speciated with organisms that typically cause disease in insects but not in mammals [7,30]. Thus, Clade D has members with a propensity to cause serious and sometimes invasive mammalian disease, although these organisms may not be as virulent as the anthrax organisms in Clade C, unless they harbor the pXO1/pXO2 plasmids. This suggests that the virulence associated with Clade D organisms, including *B. cereus *biovar *anthracis *Cl, relies not only on the virulence plasmids pXO1/pXO2, but the underlying genomic coding content shared by Clade D organisms, and that this genomic content is distinct from Clade C organisms. Clade D organisms may possess unique pathogenic traits that differentiate these organisms from *B. anthracis*.

### Genome summaries and pathway predictions from the UMd and original NCBI annotations

**Figure 3A and B:**
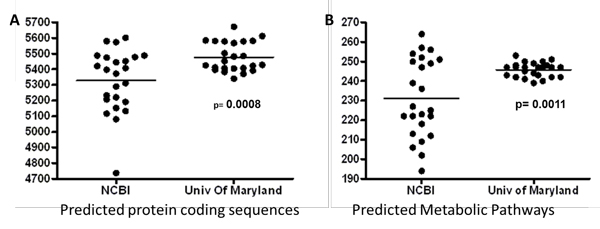
There was an increase in core proteins after reannotation. Figure 3A, The average number of protein coding sequences predicted from these genomes increased significantly with reannotation and these were statistically significant increases, p = 0.0008. Figure 3B, The predicted aggregate metabolism derived from these genome sequence annotations also was much more similar after reannotation and this was also significant p = 0.0011.

Whole genome sequences from the *B. cereus sensu lato *group that were used in this study are listed in Table 1. These genome sequences have accumulated over a 10-year period, and have been provided by a number of different sequencing centers. Due both to the differences in annotation methodology and the improvements in annotation that have occurred over the last decade, we suspected that comparisons of these annotations might be somewhat uneven. Consequently, we wanted to ensure that these genomes were annotated to a single standard. We employed the UMIGS [18] automated annotation pipeline for this process. This was an arbitrary selection, and we did not compare the annotation outputs that could have been obtained from several publicly-available annotation pipelines [31-33]. Our purpose was not to compete different annotation pipelines against one another for evaluation, but merely to ensure that these annotations met a common standard prior to subsequent analyses. Indeed, reannotation substantially changed the interpretation of these genome sequences. In general, re-annotation suggested that the protein coding content of these genomes was even more closely related than has appeared in recent analyses [10,12]. [We will exclude *B. cytotoxicus *from the remainder of this discussion, as this organism has a substantially smaller genome than the remainder of these organisms [34], and skews the interpretation of these comparisons.] The average number of protein coding sequences predicted from these genomes increased from 5268 to 5430, and the range of predicted protein coding sequences in these genomes increased from 4737-5602 (original annotations) to 5340-5613 (UMd annotations; Figure 3a); these were statistically significant increases. The predicted aggregate metabolism derived from these genome sequence annotations also was much more similar after reannotation (Figure 3b); the average number of recognizable metabolic pathways extracted from these genomes increased from 231 to 247, and the range of predicted metabolic pathways went from 194-264 to 239-265 (original vs. UMd annotations, respectively). Notably, these changes increased the estimated cluster of core proteins encoded by all 25 organisms from 1483 to 1544, an increase of 4% (Additional file 5).

There are a variety of reasons for these differences. The original annotations of these genomes accumulated over a period of ten years (Table 1), offered from nine different sequencing groups. There are currently no set guidelines or standards for genome annotation, and as a result genome annotations vary from one source to another [35]. Despite the number of annotations available in the public domain, a surprisingly large number of genes are still not annotated [36]. Missed genes have a particularly strong impact on the delineation of the core genome for a large and diverse group of organisms, and on identification of strain- or species-specific genes. The percentage of missed genes in a genome has been estimated at 5-10%, which is consistent with the collective results of this reannotation [37].

### Core genome COGS analysis, basal metabolism, and conserved hypothetical proteins amongst the clades

Results from MEGAN analysis of the core genome which comprised of the 24 and 25 membered clusters, showed there were a total of 850 genes associated with the COGs category metabolism, which was comprised of these subset of functions for the given number of genes: Energy production and conversion-137, Carbohydrate transport and metabolism-100, Amino acid transport and metabolism- 206, Nucleotide transport and metabolism-69, Coenzyme transport and metabolism-101, Lipid transport and metabolism-60, Inorganic ion transport and metabolism-139, Secondary metabolites biosynthesis, transport and catabolism-38; 461 genes were associated with information, storage and processing, which included the following subset of functions: Translation, ribosomal structure and biogenesis-140, Transcription-188, Replication, recombination and repair-132, and chromatin structure and dynamics-1; 332 genes were associated with cellular processes and signaling, which included the following subset of functions: Cell cycle control, cell division, and chromosome partitioning-25, Signal transduction mechanisms-84, Cell wall/membrane/envelope biogenesis-79, Intracellular trafficking, secretion, and vesicular transport-29, Posttranslational modification, protein turnover, and chaperones-60, Defense mechanisms-49, and cell motility-6; 344 genes were unassigned due to lack of experimental information about them, and 705 genes were assigned as poorly characterized since there was limited information available for them in order to be placed confidently into a functional category (Additional file 6 Figure 4). Since there were a large number of genes in the metabolism-related subset of functional groups, we next examined the basal metabolism in these organisms. We found that the differences in basal metabolism were slight, with only one unique enzyme, tagatose 6-phosphate kinase, found in clade A and no other unique intermediary metabolic enzymes found in clades B, C and D. However, it is unclear how this enzyme may contribute to clade A-specific phenotypes, since little work has been reported in this group of organisms. We also encountered some heterogeneity within each clade: enzymes unique to single organisms within each clade though not present in all organisms of the given clade (Additional file 7). While there is little difference between these clades in terms of intermediary metabolism, we speculate that the regulatory control of these proteins is likely different in different organisms, and could contribute to phenotypic differences between these clades, even though coding content is highly similar.

**Figure 4:**
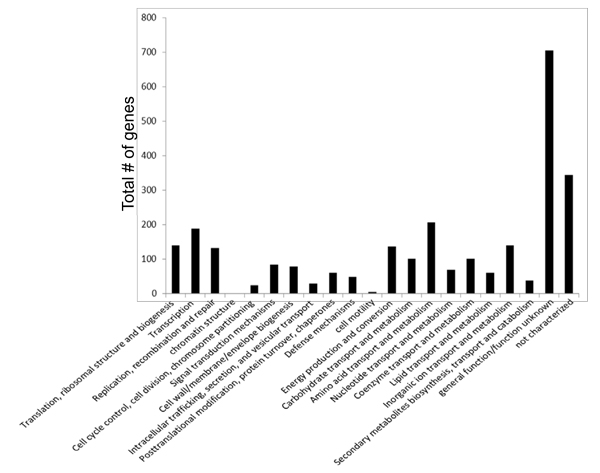
**The largest groups of genes were those with metabolism-related functions**. Subsets of clusters of orthologous genes (COGs) categories from core genes common to clades A-D. The subsets of COGs categories are shown here as functional groups as denoted by MEGAN. Genes not found by the COGS analysis tool were placed in non-characterized/function and unknown category.

We also parsed the dataset for conserved/hypothetical proteins shared by all members of a given clade while not being found in any other clade organism. With the exception of Clade C, the contribution of clade-specific conserved hypotheticals to genomic coding capacity was minimal (Additional file 8). The majority of Clade C-specific conserved hypothetical proteins appear to be contained in 4 bacteriophage lysogens that had earlier been shown to be unique to *B. anthracis *[38,39]. Five of the clade C proteins were predicted by SignalP to have a signal peptide and could therefore potentially be cell wall or secreted proteins that may be important for anthrax pathogenesis. Consequently, these conserved/hypothetical proteins may contain additional information about some of the unique biology that contributes specifically to clade C organisms. However, currently these proteins have yet to be studied and characterized therefore additional experiments which are beyond the scope of this current study would be necessary to establish a precise function for these proteins (Additional file 5).

### Comparison of whole genome protein coding content to B. cereus sensu lato sigB regulons

Strikingly, the genomic relationships between these organisms, shown in Figure 2, were identical to those that we had previously identified, based on the predicted structure of the generalized stress response regulon controlled by SigB [21]. Those observations are summarized in Table 2 for comparison. Organisms in the present Clade C appear to encode a generalized stress response, controlled by SigB, that has diverged from other members of this group to include additional functions that we hypothesized are uniquely involved in invasive disease; Clade D organisms have a SigB regulon of similar structure. By contrast, organisms in Clades A and B of the present study, many of which were associated with food poisoning outbreaks, carry a SigB regulon that either harbors only the core SigB regulon genes found in all *B. cereus sensu lato *organisms (Clade B), or have added to this core SigB regulon additional genes (Clade A) that may act enhance the stress response of these organisms during the response to deleterious environmental conditions (e.g., cold temperatures, UV light) that may be found in food processing facilities. Importantly, differences between the SigB regulon structure in these four clades are primarily a consequence of whether components of the core genome are/are not driven by a SigB promoter [21], rather than differences in gene content between these clades; the majority of genes contained in the SigB regulons of these organisms are included in the core genomes of these organisms, and their differences lie in whether their transcription is controlled by SigB. That we arrived at the same genomic relationships between these organisms using two entirely different approaches suggests an underlying organization in this population that has not been previously noted. The four clades that we identified in Figure 2 appear to be a result of at least two processes acting in parallel: 1) divergence in genomic coding capacity (protein coding genes), and 2) divergence in the generalized stress response regulon. As noted above, these processes appear to be independent of the extrachromosomal DNA content of these organisms (see Figure 2). This suggests the more general hypothesis that divergence among the members of the *B. cereus sensu lato *group relies on a pattern of regulatory divergence overlaying the protein coding divergence seen in Figure 2. Obviously, further work is necessary to test this hypothesis. In particular, does additional regulatory divergence in these organisms follow this same pattern, or is this restricted to the divergence of the SigB-controlled generalized stress response? Our recent informatics analysis (Scott EJ and Dyer DW, manuscript in preparation) suggests that the SigM ECF sigma factor regulons in these organisms appear to diverge in the same manner as we have observed here; the SigM regulons can be sorted into four clusters that also correspond to those described here. Thus, our finding that coupling protein coding divergence and regulatory divergence may be a more general phenomenon that contributes to the phenomotype of any given strain.

## Conclusion

Thus, the biological divergence in the four clades of *B. cereus sensu lato *organisms shown in Figure 2 seems to be a consequence of three evolutionary strategies that contribute in different ways. HGT of extrachromosomal elements (e.g., pXO1 and pXO2 in anthrax-like disease, the *cry *toxin plasmids in *B. thuringiensis *strains) is obviously important, but the combined forces of HGT and gene duplication/divergence acting at the genomic level appear to have promoted the divergence of these *B. cereus sensu lato *organisms into four separable clades that are not solely defined by plasmid inheritance, as illustrated in Figure 2. Previous studies have reported similar clade structures to ours [10,12,21]. However, the main differences between our study and others is that we focused solely on genomic content in order to understand the phenomotypic differences between *Bacillus anthracis *strains and other strains from the *Bacillus cereus sensu lato *group. We also utilized all chromosomal proteins (both core and unique proteins) for our study. Comparison of the draft genome clusters to the clade structure from the whole genomes heatmap, suggested similar relationships. Future studies will include strategies to determine how much of an influence the variation in annotation may have on the output of the data; perhaps this allow us select for those genomes with less noise, to enhance the comparisons of draft and whole genome datasets. Lastly, divergence of the SigB generalized stress response regulons in these organisms (summarized in Table 2) mirrors the population structure arising from bulk protein coding sequence comparisons. This suggests the hypothesis that patterns of regulatory divergence between these four clades are superimposed over the minimal differential genomic coding capacity found in these genomes. This level of divergence could fine-tune the protein expression patterns of the clade-specific gene sets, to increase fitness in specific environments. Further work to examine the divergence of transcriptional regulatory networks in these organisms is necessary to test this hypothesis.

## Availability of supporting data

"The data sets supporting the results of this article are included within the article and its additional files"

## List of abbreviations used

COGs- Clusters of Orthologous Genes, KEGGS- Kyoto Encyclopedia of Genes and Genomes, SRI- Stanford Research Institute, UMd- University of Maryland

## Competing interests

The authors declare that they have no competing interests.

## Authors' contributions

IT performed whole genome comparative analysis including pathway, metabolism, and COGS analysis. DW co-wrote this manuscript and advised on experiments to include in the paper. JW performed analysis on the conserved/hypothetical proteins and co-wrote that section of the paper.

## Supplementary Material

Additional File 1 Table S1.xls; This file contains a list of all 201 draft and whole genomes used in the study and source/location of isolation information where available. Genomes with no information available to determine source or location of isolation are denoted with "N/A". In this table, the genome names are written in the order for which they appear in Figure 1. Click here for file

Additional File 2 **Table S2**.xls; This file contains sheets A-D which show the Cd-hit clusters #0-35,000 from the Clusters of Orthologous Proteins for 201 draft and whole genomes. Click here for file

Additional File 3 **Table S3**.xls: This file contains a sheet showing Cd-hit clusters #35001-92805 from the Clusters of Orthologous Proteins for 201 draft and whole genomes. Click here for file

Additional File 4 **Table S4**.xls; This file contains Cd-hit output data from 25 whole genomes. This file contains a list of all clusters of orthologous proteins generated during our analysis. Click here for file

Additional File 5 **Table S5**.xls; This table shows number of pathways, total gene counts, average gene length and percent coding. Click here for file

Additional File 6 **Table S6**.xls; COGS categories and subset of COGS categories. Number of genes found for subset of COGs categories. Click here for file

Additional File 7 **Table S7**.xls; This table consists of parts A-D with data available in 4 spreadsheets. Each spreadsheet contains subsets of data from the KEGGs Enzyme analysis files. Also within this table are all enzymes from the KEGGs database as compared to our clade organisms. Click here for file

Additional File 8 **Table S8**.xls; Analysis of conserved/hypothetical proteins. List of conserved/hypothetical proteins genome id # with known hits in pfam, signal p, and TmHmm databases. Click here for file
